# Intermittent Ketamine and Neuromodulation Treatment Evoke Synergistic Antidepressant-Relevant Action

**DOI:** 10.1016/j.biopsych.2025.10.009

**Published:** 2025-10-17

**Authors:** Kyle A. Brown, Todd D. Gould

**Affiliations:** Department of Psychiatry, University of Maryland School of Medicine, Baltimore, Maryland (KAB, TDG); Department of Pharmacology and Physiology, University of Maryland School of Medicine, Baltimore, Maryland (TDG); Department of Neurobiology, University of Maryland School of Medicine, Baltimore, Maryland (TDG); and Veterans Affairs Maryland Health Care System, Baltimore, Maryland (TDG).

## Abstract

**BACKGROUND:**

Historically, the most effective antidepressant is electroconvulsive therapy (ECT). ECT induces cognitive side effects and commonly requires numerous treatments over several weeks for robust symptom relief. The rapid response that can be observed following ketamine treatment has led to a reconceptualization of how pharmacotherapy can treat depression. One subanesthetic infusion can alleviate symptoms within hours; however, sustained relief also requires multiple doses. Clinical studies have combined ECT and ketamine to improve therapeutic outcomes but have yielded mixed results.

**METHODS:**

We used behavioral pharmacology and ex vivo hippocampal slice electrophysiology to test the hypothesis that a preclinical ECT model, electroconvulsive stimulation (ECS), and ketamine can exert synergistic antidepressant-relevant effects in adult mice dependent on dose and dose interval.

**RESULTS:**

Repeated doses of ketamine, but not ECS, led to a metaplasticity characterized by an increased magnitude of synaptic potentiation at the hippocampal Schaffer collateral–CA1 synapse. Targeting ketamine-induced metaplasticity intermittently (i.e., 24-hour interval) by delivering individual, subeffective ketamine and ECS doses produced synergistic antidepressant-like synaptic and behavioral outcomes that resembled the actions of an effective ketamine dose. Unlike repeated ECS, intermittent ketamine and ECS administration yielded antidepressant-relevant synaptic and behavioral actions without impairing cognition. Antidepressant-like effects produced by ketamine alone were prolonged when intermittently administered with an individual ECS treatment. Synergistic effects were precluded when ketamine and ECS were delivered concurrently.

**CONCLUSIONS:**

Ketamine and ECS exhibited time-sensitive, synergistic antidepressant-like actions, suggesting that strategies targeting metaplasticity at glutamatergic synapses can be used to reduce side effects while augmenting, accelerating, and prolonging antidepressant outcomes.

Depression is a global health burden estimated to deprive the world economy of hundreds of billions of U.S. dollars annually (1). A limited ability for changes in strength at synapses responsible for regulating affect has been increasingly implicated in depression symptomology (2). The hypothesis of depression as a disorder of limited plasticity is supported by results demonstrating an impaired capacity for long-term plasticity in individuals diagnosed with depression (3). For example, the formation of cortical long-term potentiation (LTP)–like activity has been shown to be limited only when individuals diagnosed with major depressive disorder (MDD) are symptomatic (4). Thus, the use of pharmacological or neuromodulatory interventions to deliberately promote plasticity (plastogens) has led to a reconceptualization of clinical care for depression (3,5,6).

After nearly a century of clinical use, electroconvulsive therapy (ECT) remains the most effective antidepressant, including in cases of treatment resistance (7–9) [although evidence suggests that ketamine may be equally effective in subpopulations (10)]. While detailed therapeutic mechanisms of ECT are unknown, antidepressant outcomes have been linked to nonselective, long-lasting alterations in synaptic transmission (11) that then promote NMDA receptor (NMDAR)–dependent functional (12,13) and structural plasticity (7,14). However, ECT is underused for reasons including cognitive side effects and the need for numerous administrations over days to weeks to induce or sustain symptom relief (15).

In contrast to the requirement of multiple ECT sessions for the onset of antidepressant effects, (*R*,*S*)-ketamine (ketamine) can alleviate depression symptoms in some individuals just hours after one subanesthetic infusion (16). While ketamine’s rapid antidepressant mechanism is proposed to be similar to ECT’s in that both are suggested to directly alter synaptic strength shortly after administration, ketamine is hypothesized to do so transiently via increased glutamate release in regions such as the hippocampus (17–19). Consistent with the neuroplasticity hypothesis of depression, rapid changes in synaptic strength observed following a single ketamine dose putatively sustain therapeutic effects by promoting NMDAR activation–dependent plasticity (3,20). However, similar to ECT, repeated ketamine infusions are required for robust, durable effects (21,22).

As ECT and ketamine appear to converge on NMDAR-dependent plasticity to produce antidepressant actions, clinicians have endeavored to improve therapeutic outcomes by combining these interventions. Some studies have found enhanced effects when ECT and ketamine are delivered concurrently (23–29), but meta-analyses of these investigations have concluded that simultaneous administration elicits no further meaningful benefit (30–33). Emerging evidence suggests that individuals diagnosed with depression who exhibited an inadequate response to ECT or ketamine/esketamine alone showed an antidepressant response, or had an earlier onset of symptom remission, when ECT and ketamine were delivered intermittently (i.e., hours to days between one another) (34–38), but additional studies are needed to validate these conclusions as they derive from case reports and underpowered studies.

Mixed therapeutic results following combined ketamine and ECT administration motivated us to ask whether ketamine and ECT’s ability to produce more robust antidepressant outcomes after multiple treatments results from persistent changes in the ability for plasticity [i.e., metaplasticity (39) or synaptic priming (40)]. We delivered ketamine or the preclinical equivalent of ECT, electroconvulsive stimulation (ECS), concurrently or intermittently to test the hypothesis that these treatments can exert synergistic antidepressant-relevant actions if dose and dose interval are optimized.

## METHODS AND MATERIALS

### Animals

Eight-week-old female CD-1 (Charles River) mice were housed in groups of 4/cage with a 12-hour light/dark cycle (lights on: 7 AM). Mice experienced no environmental enrichment [e.g., no variation in environment or diet, single-sex housing, no opportunity for exercise, no material enrichment (41,42)]. Animals acclimated to the vivarium for ≥1 week. Food and water were provided ad libitum. Experiments were approved by the University of Maryland Baltimore Institutional Animal Care and Use Committee. As rates of depression are higher in those whose biological sex is female (43), only female mice were used.

### Materials

(*R,S*)-ketamine hydrochloride (Sigma-Aldrich) was dissolved in normal saline. All other chemicals used were of analytical or higher grade (Sigma-Aldrich).

### Treatment Delivery

Mice were randomly assigned to treatment groups and received vehicle or ketamine (intraperitoneal, 7.5 mL/kg) by a male experimenter at 9 AM. An Ugo Basile pulse generator (#57800) was used to deliver ECS to mice via bilateral auricular stimulation with AISI 303 stainless steel electrodes (#57800–002; Ugo Basile) using parameters established to model ECT antidepressant-relevant outcomes [100 Hz, 0.3-second pulse width, 1-second stimulation duration, 50-mA current (44)]. As isoflurane has been shown to evoke antidepressant-relevant plasticity in the mouse hippocampus (45), mice received inhaled halothane anesthesia before or during ECS. ECS-induced tonic-clonic convulsions were consistent between mice; all mice experienced a seizure upon ECS delivery, and all survived the procedure. Sham ECS consisted of identical handling and anesthesia without stimulation. Naïve mice did not experience anesthesia and remained unhandled until behavioral experiments.

### Behavior

#### Passive Avoidance.

Hippocampal-dependent learning and memory was assessed in a 2-phase passive avoidance test as previously described (46). In the training phase, mice were individually placed in the right compartment and allowed to explore for 3 seconds, and a guillotine door opened, allowing access to the left compartment. Upon entering the left compartment, a single inescapable foot shock was delivered 3 seconds later (0.6 mA, 2-second duration). The retention phase was completed 24 hours later. An identical procedure was followed, except no shock was administered. Latency to cross was quantified.

#### Tail Suspension Test.

The antidepressant-relevant action of ketamine and ECS in the tail suspension test (TST) was completed as previously described (47). Mice were individually tested for mobility in suspension boxes over a 6-minute interval. A digital camera recorded mouse mobility while suspended by the tail.

### Electrophysiology

Hippocampal slice preparation and electrophysiology were conducted as previously described (20,48). Briefly, 400-μm transverse slices were cut in ice-cold, dissection artificial cerebrospinal fluid (ACSF) (120 mM NaCl, 3 mM KCl, 4 mM MgCl_2_, 1 mM NaH_2_PO_4_, 26 mM NaHCO_3_, 10 mM glucose, 95% O_2_/5% CO_2_). Slices recovered in a holding chamber (20–22 ° C) for >90 minutes in recording ASCF (120 mM NaCl, 3 mM KCl, 1.5 mM MgCl_2_, 1 mM NaH_2_PO_4_, 2.5 mM CaCl_2_, 26 mM NaHCO_3_, 10 mM glucose, 95% O_2_/5% CO_2_). Recordings were completed in a submersion-type chamber under continuous ACSF perfusion (1.5 mL/min; Cole-Parmer).

Schaffer collaterals (SCs) were stimulated with a bipolar electrode (FHC). ACSF-filled glass recording pipettes (3–5 MΩ) recorded a field excitatory postsynaptic potential (fEPSP) in the CA1 stratum radiatum. An input/output (I/O) curve determined basal synaptic strength at 10 stimulus intensities (75–300 μA, 20-second interval). The stimulus intensity was modified to elicit 35% to 50% of the maximal fEPSP slope, and paired-pulse fEPSPs (50 ms; paired-pulse ratio [PPR]) were recorded. Single-pulse fEPSPs were then monitored for 10 minutes. High-frequency stimulation (HFS) induced LTP (4 × 100 Hz/1-second train, 20-second interval).

Data were analyzed with pCLAMP software (Molecular Devices). The fEPSP was normalized to the average slope recorded during 6 to 10 minutes. If the average slope during 1 to 5 minutes exhibited >10% variation, the slice was excluded. LTP magnitude reflects the average value during 56 to 60 minutes post-HFS.

### Statistics

Experiments were performed and analyzed by a blinded experimenter. Experiments with 1 factor containing 2 levels were analyzed with 2-tailed Student’s *t* tests. Experiments with 1 factor with >2 levels were analyzed via 1-way analysis of variance (ANOVA). Two-way ANOVA analyzed experimental results with >1 factor. I/O curves and passive avoidance performance were analyzed with 1-way or 2-way repeated-measures (RM) ANOVA, as appropriate. Treatment means were separated via the Holm-Šídák test. Homoscedasticity (Brown-Forsythe test) and normality (Shapiro-Wilk test) were assessed. Data was log transformed when not normally distributed. Two-way RM ANOVA with a factor containing >2 levels was conducted in RStudio. Other analysis was completed in GraphPad Prism. *n* Values indicate the number of mice assessed. An α level of 0.05 was used as the criterion for statistical significance. Details on sample sizes, statistical tests, and statistical outcomes are provided in Table S1

## RESULTS

For full details of statistical outcomes, see Table S1.

### Repeated, Intermittent Ketamine Doses Induce an Increase in the Ability for Hippocampal Synaptic Potentiation Compared With a Single Dose

**Figure 1.**
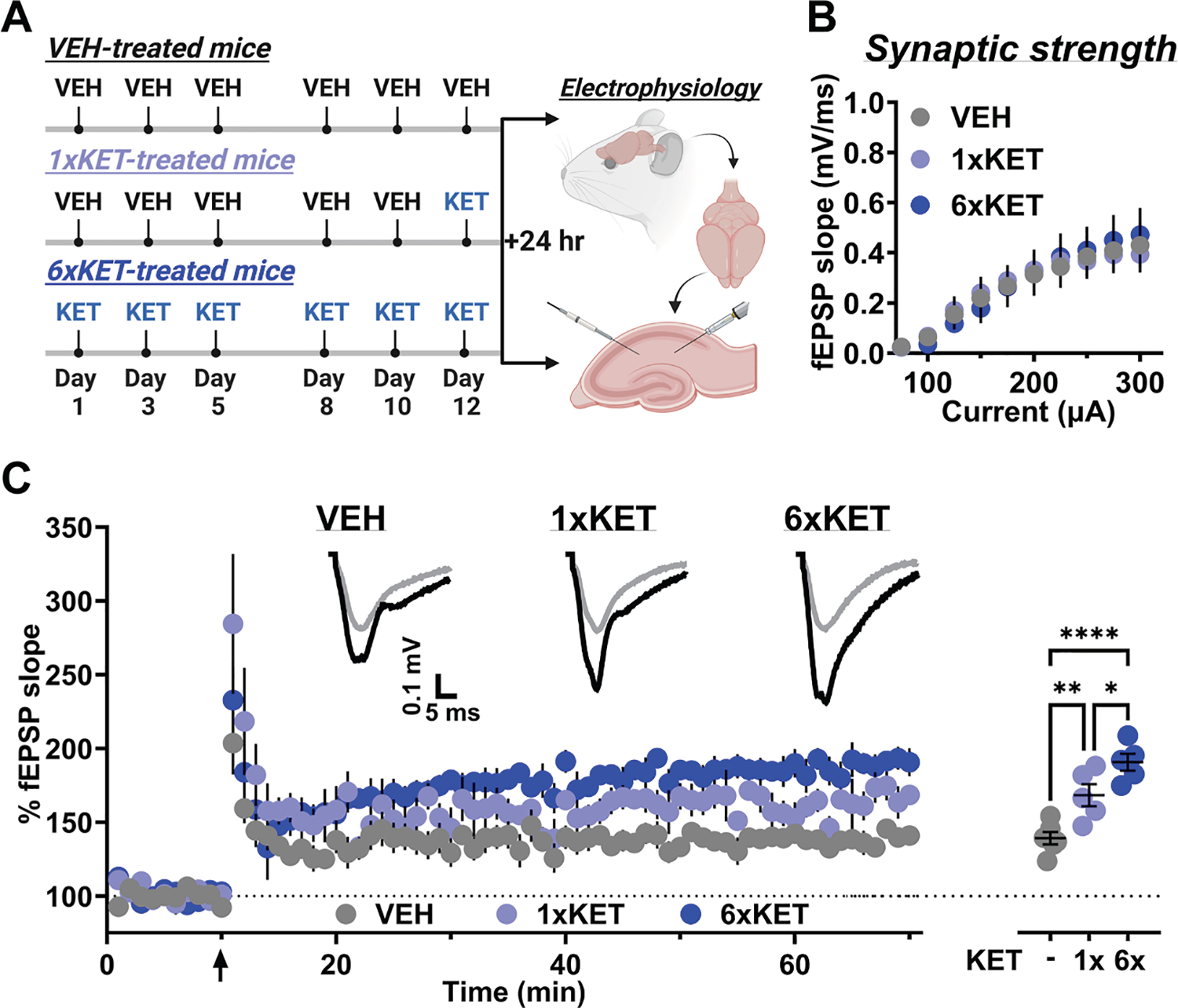
Repeated, intermittent KET doses induce an increase in the ability for hippocampal synaptic potentiation compared with a single dose. **(A)** To test whether a single dose of KET primes plasticity evoked by another dose, mice received intraperitoneal injections of VEH, 10 mg/kg KET once (1× KET), or 6 times in a schedule that resembles clinical infusion paradigms (6× KET). Mice were euthanized and hippocampal slices were collected 24 hours after the last injection for electrophysiology experiments in which fEPSPs were recorded from the hippocampal Schaffer collateral–CA1 synapse. **(B)** KET had no prolonged effect on basal synaptic strength as revealed by the unaltered input/output relationship. **(C)** Long-term potentiation magnitude was enhanced in slices collected from mice 24 hours after 1× KET administration compared with VEH-treated mice. This enhancement was significantly augmented in 6× KET-treated mice compared with VEH-treated and 1× KET–treated mice. Arrow at *t* = 10 minutes denotes 4× 100-Hz high-frequency stimulation. Traces are composed of representative sweeps from 5 minutes pre-tetanus (gray) and 56 to 60 minutes post-tetanus (black). Data represent mean ± SEM. *p < .05, **p < .01, ****p < .0001 as indicated by Holm-Šídák post hoc comparisons. See Table S1 for complete details on the statistical analyses and precise group sizes. fEPSP, field excitatory postsynaptic potential; KET, (*R*,*S*)-ketamine; VEH, vehicle.

To determine whether repeated ketamine doses uniquely alter plasticity compared with a single dose, we assessed the ability for LTP 24 hours after the last of either a single or repeated ketamine treatments. We assessed synaptic plasticity at a hippocampal synapse implicated in both depression phenotypes and antidepressant-relevant plasticity, SC-CA1 (2) (Figure 1A). The intermittent dosing schedule (a vehicle or ketamine dose on Monday, Wednesday, and Friday for 2 weeks) was designed to mimic common clinical infusion regimens (10). Animals were euthanized 24 hours after the last dose as clinical studies have reported maximal antidepressant effects following a single ketamine infusion at this time (16). Preclinical studies also demonstrate synaptic metaplasticity at SC-CA1 24 hours postdose (3). We used a dose (10 mg/kg) established to produce a pharmacodynamic profile in mice that is consistent with the action of antidepressant infusion in humans (49).

Twenty-four hours after the last dose, the I/O relationship of the fEPSP revealed that basal synaptic strength was unaffected by 1 or 6 ketamine doses (Figure 1B). The fEPSP PPR, an indicator of short-term presynaptic plasticity that indicates short-term (<3 hours), but not long-term (>3 hours), synaptic action of ketamine metabolites (48), was also unchanged (Figure S1A). There was a significant enhancement in the ability for HFS-evoked synaptic potentiation in slices collected from mice treated with a single ketamine dose compared with mice that received vehicle (Figure 1C). LTP magnitude was significantly augmented in slices derived from mice that received 6 ketamine injections compared with mice that received 1. As no change in basal synaptic strength was measured in slices from ketamine-treated mice, these results suggest that, rather than directly and persistently potentiating SC-CA1, the initial dose(s) of ketamine induces a metaplasticity that is acted on by later doses to result in synapses being more amenable to electrically evoked synaptic potentiation.

### Repeated Intermittent ECS Results in Cognitive Impairment and Antidepressant-Relevant Synaptic and Behavioral Outcomes

**Figure 2.**
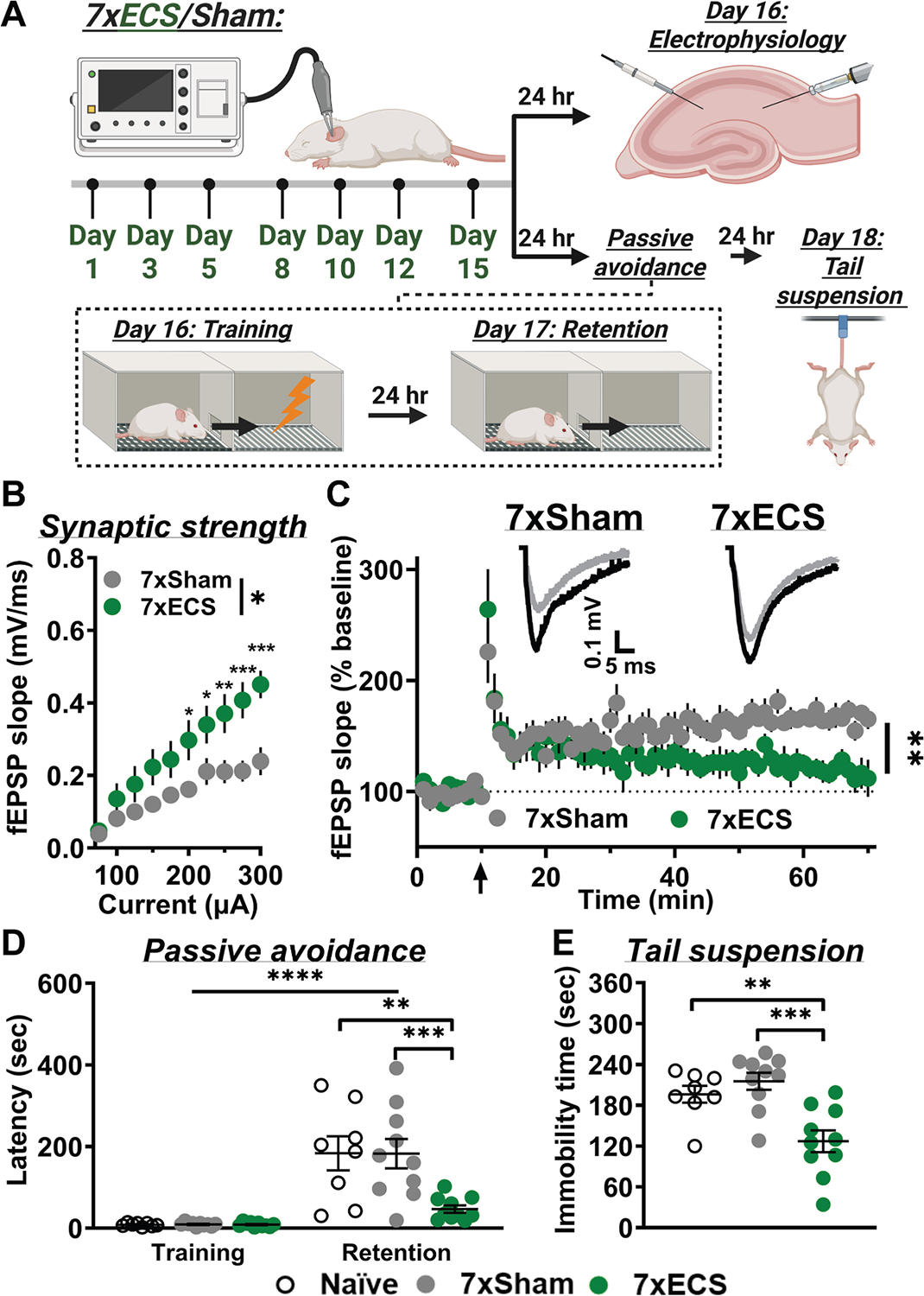
Repeated intermittent ECS results in cognitive impairment and antidepressant-relevant synaptic and behavioral outcomes. **(A)** To test whether repeated, intermittent ECS administrations evoke antidepressant-relevant synaptic and behavioral outcomes, mice received 7 total ECS or sham ECS under halothane anesthesia over 15 days. Mice were euthanized and hippocampal slices were collected 24 hours later (day 16) for electrophysiology experiments in which field excitatory postsynaptic potentials were recorded from the hippocampal SC-CA1 synapse. Mice euthanized for electrophysiology were not used for behavioral experiments. A separate cohort of mice treated with repeated sham or ECS was assessed for cognitive impairment 24 hours later in the passive avoidance task (days 16–17). This cohort of mice was then assessed for antidepressant-like behavior in the TST 72 hours after the last sham or ECS (day 18). Mice that were naïve to sham or ECS experience were included in behavioral studies to confirm that sham conditions did not significantly alter performance in the tasks. **(B)** Basal synaptic strength at the SC-CA1 synapse was significantly potentiated in slices derived from mice treated with repeated ECS compared with repeated sham. **(C)** The ability for LTP was significantly impaired in slices derived from mice that received repeated ECS. Traces are composed of representative sweeps from 5 minutes pre-tetanus (gray) and 56 to 60 minutes post-tetanus (black) for each treatment group. Arrow at *t* = 10 minutes denotes 4× 100-Hz high-frequency stimulation. LTP magnitude recorded from each animal is presented in Figure S2A. **p < .01 as indicated by unpaired Student’s *t* test. **(D)** Mice treated with repeated ECS or sham ECS (as well as mice naïve to both ECS or sham ECS conditions) learned and remembered in the passive avoidance task, but ECS-treated mice performed significantly worse in the retention phase than sham-treated and naïve mice. Performance within each phase of the task is depicted in Figure S2B, C. **(E)** ECS-treated mice were significantly more mobile in the TST than naïve and sham-treated mice, indicating an antidepressant-like effect of repeated ECS. Data represent mean ± SEM. *p < .05, **p < .01, ***p < .001, ****p < .0001 as indicated by Holm-Šídák post hoc comparisons. See Table S1 for complete details on the statistical analyses and precise group sizes. ECS, electroconvulsive stimulation; LTP, long-term potentiation; SC, Schaffer collateral; TST, tail suspension test.

To compare the synaptic and behavioral actions of repeated ketamine treatments with multiple administrations of antidepressant neuromodulation, we measured the effect of repeated, intermittent sham or ECS treatments 24 to 72 hours after the last of 7 total administrations (Figure 2A). The quantity and interval of ECS were designed to mimic intermittent schedules commonly used in clinical stimulation regimens [e.g., 2–3 times weekly over 2 to 3 weeks (10,15)].

Twenty-four hours after the last of 7 sham ECS or ECS, slices derived from ECS-treated mice exhibited increased SC-CA1 basal synaptic strength (Figure 2B). No change in PPR was detected (Figure S1B). Slices collected from ECS-treated mice were unable to express and maintain LTP (Figure 2C; Figure S2A), consistent with LTP occlusion.

Next, we assessed whether repeated ECS impaired mouse performance in an SC-CA1 synapse-dependent learning and memory test, the passive avoidance task (50) (Figure 2D). Naïve, sham-treated, and ECS-treated mice performed similarly in passive avoidance training (Figure 2D; Figure S2B). Twenty-four hours after training, no significant difference in performance in the retention phase was detected between sham-treated and naïve mice; however, it took ECS-treated animals significantly less time to cross into the compartment they were shocked in, consistent with impaired cognition (Figure 2D; Figure S2C). To evaluate antidepressant-relevant behavioral effects of our ECS protocol, we assessed the performance of mice in a classic test of antidepressant efficacy, the TST, 72 hours after the last ECS or sham event. Compared with sham-treated mice, animals that received repeated doses of ECS spent significantly more time mobile in the TST, consistent with a sustained antidepressant-like effect (Figure 2E). Animal body weight was unaffected by repeated ECS (Figure S2D).

### Concurrent Ketamine and ECS Administration Induces No Synergistic Antidepressant-Relevant Synaptic or Behavioral Effects

**Figure 3.**
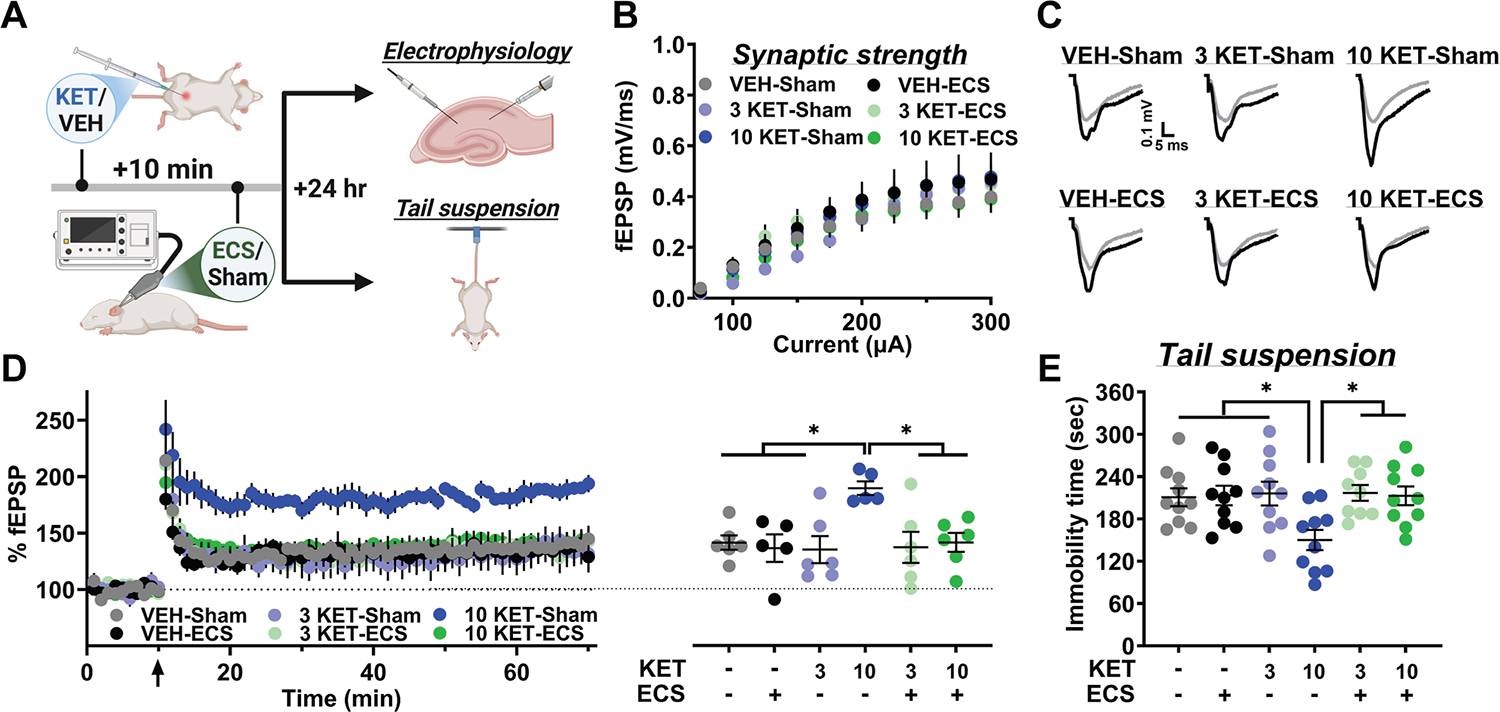
Concurrent ketamine and ECS administration induces no synergistic antidepressant-relevant synaptic or behavioral effects. **(A)** To test whether a single dose of KET delivered concurrently with ECS induces synergistic antidepressant-like action, mice received an injection of VEH, 3 mg/kg KET (3 KET), or 10 mg/kg KET (10 KET) 10 minutes before sham ECS or ECS delivery under halothane anesthesia. Mice were euthanized and hippocampal slices were collected 24 hours later for electrophysiology experiments in which fEPSPs were recorded from the hippocampal Schaffer collateral–CA1 synapse. Mice euthanized for electrophysiology experiments were not used for behavioral experiments. A separate cohort of mice was assessed for antidepressant-relevant behavior in the TST 24 hours after the ECS or sham ECS. Separate cohorts of mice were used for the TST and passive avoidance tasks. **(B)** KET and/or ECS had no prolonged effect on basal synaptic strength as revealed by the unaltered input/output relationship. **(C, D)** Compared with all other groups, long-term potentiation magnitude was enhanced only in slices derived from 10 KET-sham–treated mice. Traces are composed of representative sweeps from 5 minutes pre-tetanus (gray) and 56 to 60 minutes post-tetanus (black) for each treatment group. Arrow at *t* = 10 minutes denotes 4× 100-Hz high-frequency stimulation. **(E)** Consistent with a KET-evoked antidepressant-relevant plasticity, 10 KET-sham mice were significantly more mobile in the TST than all other treatment groups. Data represent mean ± SEM. *p < .05 as indicated by Holm-Šídák post hoc comparisons. See Table S1 for complete details on the statistical analyses and precise group sizes. ECS, electroconvulsive stimulation; fEPSP, field excitatory postsynaptic potential; KET, (*R*,*S*)-ketamine; TST, tail suspension test; VEH, vehicle.

To evaluate the antidepressant-relevant actions of concurrent ECS and ketamine delivery, we administered vehicle or ketamine 10 minutes before either a single sham or ECS. Synaptic and behavioral changes were assessed 24 hours later (Figure 3A). The duration before ECS approximates the time ketamine takes to reach maximal concentration in the mouse brain after an intraperitoneal injection (49). Animals received either vehicle, 10 mg/kg ketamine, or a ketamine dose that we have previously shown fails to evoke rapid and sustained antidepressant-relevant effects on its own (i.e., 3 mg/kg [defined as a subeffective dose]) but produces synergistic outcomes when combined with drugs that elicit rapid and sustained antidepressant-like effects through putative, ECS-relevant actions involving potentiation of glutamatergic activity (20).

Twenty-four hours after administration, slices derived from mice that received ketamine or ECS exhibited no significant change in basal synaptic strength compared with slices from mice that received vehicle or sham treatments (Figure 3B). No change in the I/O response in slices from mice that received a single ECS is consistent with a subthreshold ECS dose in the context of its antidepressant-relevant outcomes. PPR was unchanged by ketamine or ECS treatment (Figure S1C). When delivered 10 minutes before sham ECS, 3 mg/kg ketamine did not alter LTP magnitude, whereas a 10-mg/kg dose significantly enhanced LTP magnitude (Figure 3C, D). This effect was reversed when 10 mg/kg ketamine was combined with ECS. No additive or synergistic effects of 3 mg/kg ketamine combined with ECS were detected. In a separate cohort, mice treated with 10 mg/kg ketamine and sham ECS were significantly more mobile compared with all other treatment groups in the TST, indicating that ECS blocks antidepressant-relevant behavior when delivered concurrently with ketamine (Figure 3E).

### Single, Intermittent Deliveries of Ketamine and ECS Induce Synergistic Antidepressant-Relevant Outcomes

**Figure 4.**
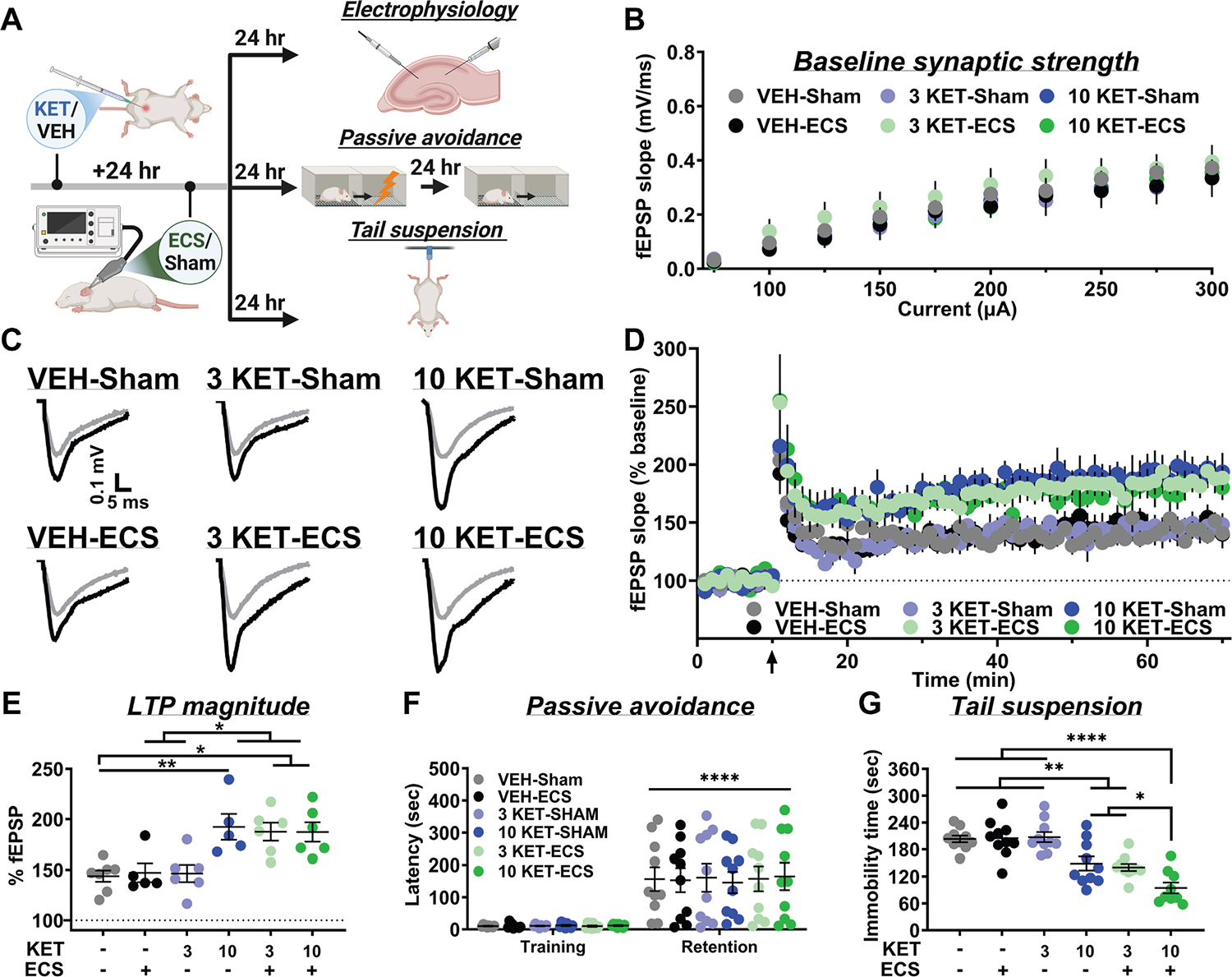
Single, intermittent deliveries of ketamine and ECS induce synergistic antidepressant-relevant outcomes. **(A)** To test whether a single dose of KET can prime plasticity such that ECS delivered the next day evokes synergistic antidepressant-like effects, mice received an injection of VEH, 3 mg/kg KET (3 KET), or 10 mg/kg KET (10 KET) 24 hours before sham ECS or ECS administration under halothane anesthesia. Twenty-four hours after sham or ECS, mice were tested in the passive avoidance task, TST, or were euthanized and hippocampal slices were collected for electrophysiology experiments in which fEPSPs were recorded from the hippocampal Schaffer collateral–CA1 synapse. Mice euthanized for electrophysiology experiments were not used for behavioral experiments. Separate cohorts of mice were used for the TST and passive avoidance tasks. **(B)** KET and/or ECS had no prolonged effect on basal synaptic strength. **(C–E)** Mice that received a dose of KET insufficient to prime LTP on its own (3 mg/kg; 3 KET-sham) evoked a response that resembled the effect of a threshold dose (10 mg/kg; 10 KET-sham) when administered 24 hours before ECS (3 KET-ECS). 10 KET-ECS had a similar enhancing effect on LTP as 10 KET-Sham and 3 KET-ECS. Arrow at *t* = 10 minutes denotes high-frequency stimulation. Traces are composed of representative sweeps from 5 minutes pre-tetanus (gray) and 56 to 60 minutes post-tetanus (black) for each treatment group. **(F)** Unlike after repeated ECS (Figure 2D), a single ECS delivered intermittently with VEH/KET did not alter learning and memory in the passive avoidance task. Performance within each phase of the task is depicted in Figure S3. **(G)** Mice treated with 10 KET-sham exhibited decreased immobility time in the TST, an effect that was mirrored by 3 KET-ECS–treated mice. This outcome was augmented in 10 KET-ECS–treated mice. Data represent mean ± SEM. *p < .05, **p < .01, ****p < .0001 as indicated by Holm-Šídák comparisons. Table S1 contains complete details on statistical analyses and group sizes. ECS, electroconvulsive stimulation; fEPSP, field excitatory postsynaptic potential; KET, (*R*,*S*)-ketamine; LTP, long-term potentiation; TST, tail suspension test; VEH, vehicle.

To evaluate the antidepressant-relevant actions of intermittent ECS and ketamine, vehicle or ketamine was delivered 24 hours before sham or ECS to allow sufficient time for ketamine elimination (49) and the activation of metaplastic mechanisms (3) (Figure 4A). Mice completed behavioral tests or were euthanized for electrophysiology experiments 24 hours following sham or ECS (48 hours post-vehicle/ketamine).

Basal SC-CA1 synaptic strength (Figure 4B) and PPR (Figure S1D) were unchanged by treatment conditions. Strikingly, a 3-mg/kg dose of ketamine administered 24 hours before ECS enhanced LTP magnitude in a manner that mirrored the priming effect of 10 mg/kg ketamine-sham ECS (Figure 4C–E). The 10-mg/kg dose administered 24 hours pre-ECS did not augment LTP magnitude further, indicating a ceiling effect. Increased LTP magnitude remained detectable 48 hours after a 10-mg/kg ketamine dose when delivered 24 hours before sham ECS.

To evaluate whether electrophysiological indicators of synergistic, antidepressant-relevant metaplasticity coincide with behavioral readouts of impaired cognition or antidepressant efficacy, we assessed the performance of a separate cohort of mice in the passive avoidance task and TST (Figure 4F, G). Similar to results following either repeated ECS or sham treatment (Figure 2D), all groups performed similarly in the passive avoidance training phase (Figure 4F; Figure S3A); however, unlike mice that received repeated ECS (Figure 2D), single, intermittent delivery of ketamine and ECS did not impair retention (Figure 4F; Figure S3B). Furthermore, a separate cohort of mice that received intermittent 3 mg/kg ketamine and ECS exhibited a significant reduction in immobility time in the TST (Figure 4G). This effect resembled that of 10 mg/kg ketamine administered 24 hours before sham ECS. The decrease in immobility time was significantly augmented when 10 mg/kg ketamine was injected 24 hours pre-ECS.

### Antidepressant-Relevant Plasticity and Behavioral Outcomes Are Prolonged Following Single, Intermittent Deliveries of Ketamine and ECS

**Figure 5.**
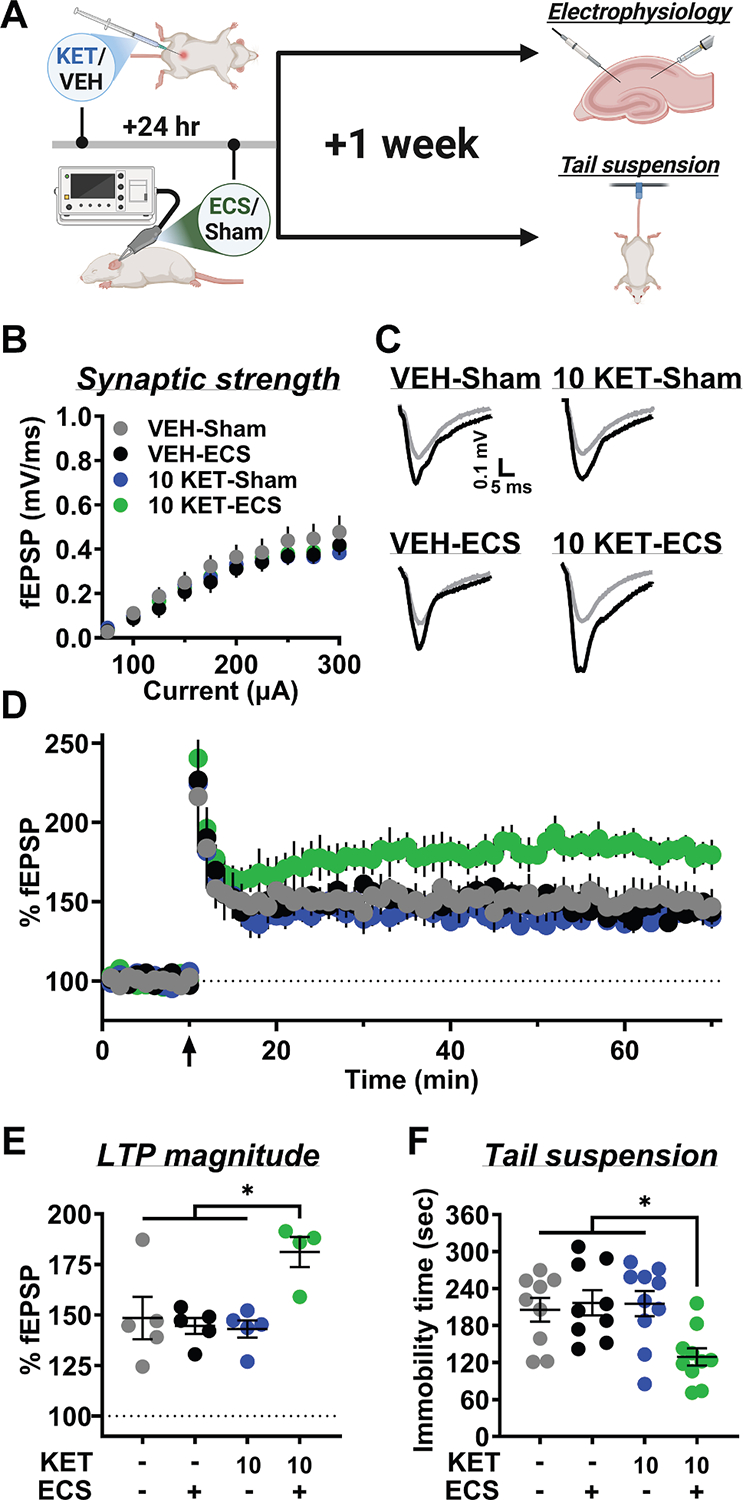
Antidepressant-relevant plasticity and behavioral outcomes are prolonged following single, intermittent deliveries of ketamine and ECS. **(A)** To test whether a single, antidepressant-relevant dose of KET can prime synaptic plasticity such that ECS delivered the next day can prolong antidepressant-like effects, mice received an injection of VEH or 10 mg/kg KET (10 KET) 24 hours before sham ECS or ECS administration under halothane anesthesia. One week after sham or ECS, mice were tested in the TST or were euthanized and hippocampal slices were collected for electrophysiology experiments in which fEPSPs were recorded from the hippocampal Schaffer collateral–CA1 synapse. Mice euthanized for electrophysiology experiments were not used for behavioral experiments. **(B)** KET and/or ECS had no prolonged effect on basal synaptic strength as revealed by the unaltered input/output relationship. **(C–E)** Enhanced LTP was no longer observed 1 week after sham ECS in slices derived from 10 KET-sham–treated mice, but increased LTP persisted in slices collected from 10 KET-ECS–treated mice. Traces are composed of representative sweeps from 5 minutes pre-tetanus (gray) and 56 to 60 minutes post-tetanus (black) for each treatment group. Arrow at *t* = 10 minutes denotes 4× 100-Hz high-frequency stimulation. (F) Consistent with prolonged antidepressant-relevant plasticity evoked by intermittent KET and ECS delivery, 10 KET-ECS–treated mice were significantly more mobile in the TST than mice in all other treatment groups 1 week after ECS. Data represent mean ± SEM. *p < .05 as indicated by Holm-Šídák post hoc comparisons. See Table S1 for complete details on the statistical analyses and precise group sizes. ECS, electroconvulsive stimulation; fEPSP, field excitatory postsynaptic potential; KET, (*R*,*S*)-ketamine; LTP, long-term potentiation; TST, tail suspension test; VEH, vehicle.

To test whether intermittent delivery of ketamine and ECS prolongs antidepressant-like plasticity and behavior, mice received either vehicle or 10 mg/kg ketamine, received sham or ECS 24 hours later, and were used for electrophysiology and behavioral experiments 1 week later (Figure 5A). This time point was selected as clinical studies have found that antidepressant benefits following a single ketamine infusion generally fade within a week (21).

One week after sham or ECS, no significant differences in basal synaptic strength (Figure 5B) or PPR (Figure S1E) were detected. LTP magnitude measured from slices derived from animals treated with 10 mg/kg ketamine-sham ECS was comparable to that of vehicle-sham–treated and vehicle-ECS–treated mice, indicating that synaptic metaplasticity had subsided (Figure 5C–E). Strikingly, the magnitude of LTP measured in slices from mice that received ECS 24 hours after 10 mg/kg ketamine remained significantly increased compared with all other treatment conditions, suggesting that intermittent delivery of ketamine and ECS can prolong antidepressant-relevant plasticity. Consistent with these electrophysiology results, mice from a separate cohort that were treated with 10 mg/kg ketamine 24 hours before ECS were significantly more mobile in the TST compared with all other treatment groups (Figure 5F).

## DISCUSSION

### Hippocampal Synaptic Metaplasticity Exerted by Repeated, Intermittent Ketamine Doses

Ketamine is typically administered in intermittent, sub-anesthetic doses in clinical care for depression (e.g., 1 daily dose 2–3 times weekly for induction) as therapeutic outcomes persist longer after serial administrations (22,51–53). Improved therapeutic actions of repeated, intermittent ketamine may result from initial infusions exerting a synaptic metaplasticity that subsequent doses harness to maintain symptom relief (3). Clinical evidence supports this, highlighted by ketamine increasing visually evoked, cortical LTP-like activity in individuals diagnosed with MDD 3 to 4 hours postinfusion (54). Similarly, preclinical reports have demonstrated that a single subanesthetic ketamine dose increases electrically induced LTP magnitude at SC-CA1 3 to 24 hours postadministration (20,55–59). Bath-applied ketamine potentiates SC-CA1 synaptic strength significantly more in hippocampal slices derived from mice treated with ketamine 7 days earlier than slices collected from vehicle-treated mice (60), suggesting that initial ketamine treatment persistently increases the ability for metaplasticity exerted by a later treatment. However, no study has directly compared the plasticity evoked by a single ketamine treatment with that elicited by multiple, intermittent doses. Here, we addressed this knowledge gap and found that slices from mice that received repeated, intermittent doses exhibited an even greater magnitude of SC-CA1 LTP compared with slices from mice that were administered 1 dose.

### The Antidepressant-Like Actions of Concurrent Ketamine and ECS

Many individuals diagnosed with depression respond inadequately to ketamine and ECT, and patients are left with few options if they remain unresponsive to both (61,62). Therefore, many studies have assessed whether concurrent delivery of ketamine and ECT can improve antidepressant response, and some of these studies have found positive results (23–29). However, meta-analyses of clinical studies combining ketamine and ECT have led to the conclusion that simultaneous administration did not enhance therapeutic response (30–33). These findings are consistent with preclinical reports that found that blocking the NMDAR via ketamine anesthesia when rats received ECS prevented antidepressant-relevant behaviors and synaptic changes measured in vivo in the dentate gyrus (12).

Here, we also observed that concurrent ketamine and ECS delivery did not produce improved effects, consistent with NMDAR antagonism at the time of ECS administration hindering antidepressant-relevant actions. Furthermore, no behavioral or synaptic effects were detected when ketamine was concurrently administered with ECS. The spatiotemporal characteristics of plasticity stimuli influence their sustained functional effects. For example, the interval between plasticity events and the ability for subsequent synaptic potentiation follows an inverted U–shape wherein excessively brief plasticity intervals [in some instances, hours after in vivo heterosynaptic plasticity induction (63)] can yield synaptic depotentiation (64–66). As administration of ketamine or ECS evokes in vivo heterosynaptic plasticity resembling the effect of standard LTP induction protocols (17,67), our observations are also consistent with the simultaneous administration of 10 mg/kg ketamine and ECS reversing plasticity and precluding synergistic antidepressant-like actions.

### Pharmacological Metaplasticity Primes the Antidepressant-Relevant Action of ECS

Emerging evidence from clinical case studies suggests that patients diagnosed with depression who were unresponsive to individually administered ketamine/esketamine or ECT respond when both are delivered intermittently (34–37). A small, double-blind, placebo-controlled trial also reported a trend indicating that patients diagnosed with treatment-resistant depression who received intermittent ketamine and ECT showed an earlier onset of symptom remission than patients who received midazolam and ECT (38). Our results are consistent with these clinical reports. We extended our finding of initial ketamine doses exerting a cumulative, persistent metaplasticity to discover that weak metaplastic priming induced by a subeffective ketamine dose (3 mg/kg) could be harnessed by ECS delivered the next day to produce synaptic and behavioral responses that mimicked the effects of an effective ketamine dose. It is noteworthy that 10 mg/kg ketamine administered a day before ECS augmented mobility in the TST but not SC-CA1 LTP magnitude. This suggests that antidepressant-relevant behavior remained sensitive to the lowered threshold for plasticity at this time point, but the electrophysiology measurement reached a ceiling, potentially resulting from the strong tetanic protocol. The magnitude of SC-CA1 LTP and antidepressant-relevant behavior were both increased a week after intermittent 10 mg/kg ketamine and ECS administration but not in mice that received 10 mg/kg ketamine and sham ECS.

### Synergistic, Antidepressant-Like Effects of Ketamine and ECS Without Impaired Cognition

Cognitive side effects are a common reason for ECT discontinuation (7). Prolonged potentiation of basal synaptic strength following repeated ECS or ECT has been linked to the intervention’s effects on depression symptoms and cognition, the latter putatively resulting from a reduced ability for LTP (12,13,68). The ECT model used here impaired SC-CA1 LTP and enhanced basal synaptic strength. These ECS-induced synaptic changes coincided with impaired cognition and improved antidepressant-like behavior. Importantly, mice treated with a subeffective or effective dose of ketamine a day before a single ECS exhibited antidepressant-relevant plasticity and behavior without a persistent increase in basal synaptic strength or impaired cognition. That is, in contrast to repeated ECS doses exerting antidepressant-relevant effects by persistently increasing basal synaptic efficacy, which also limits learning/memory, a single ketamine dose primes synaptic plasticity such that an individual ECS dose can then elicit antidepressant-relevant actions. While requiring assessment in clinical settings, the ability to produce effective antidepressant-like actions with subeffective doses of ketamine and ECS suggests an opportunity to expand access to these therapeutics for conditions in which ECT usage must be carefully considered, such as in individuals diagnosed with depression who are pregnant (69), elderly individuals who are at elevated risk of side effects from general anesthesia (70), or populations that have excellent ECT response rates but are also at high risk for side effects [e.g., patients diagnosed with depression with co-occurring psychotic features (61)].

### Strengths and Limitations

The translation of metaplasticity principles has led to a reconceptualization of how repeated, noninvasive brain stimulation can more rapidly and effectively treat depression (71). Similarly, tenets of synaptic plasticity have been used in preclinical studies to pharmacologically extend ketamine’s antidepressant-like actions (72). However, such an approach combining pharmacotherapy and ECT or ECS remains unrealized. In this study, we endeavored to directly address this knowledge gap. A strength of our report is that experiments were designed to reveal that the spatiotemporal-dependent pharmacological properties of therapeutic plastogens can be exploited to augment, accelerate, and prolong antidepressant-relevant outcomes. Our results support the use of strategies that harness metaplastic processes to improve dosage regimens, which could be applied to available antidepressants and emerging antidepressants that also prime synaptic plasticity [e.g., ketamine metabolites, psychedelics, NMDAR modulators (3,48,73–75)]. For example, the synergistic effects of intermittent ketamine and ECS are consistent with an accelerated onset of antidepressant action, which may be particularly useful for addressing depression cases with acute risk of suicidality. Longer-lasting antidepressant-relevant effects following intermittent delivery of ketamine and ECS indicate that synaptic metaplasticity can also be used to decrease the quantity and frequency of dosing, thereby reducing the burden placed on patients and health care providers.

However, the current results should be interpreted while weighing study limitations. Only female outbred CD-1 mice were used in our experiments. Future studies should also assess whether the synergistic actions detected here are observed in male mice and in inbred mouse strains that have less genetic variability [although see (76,77)]. Whether ketamine primes ECS within preclinical chronic stress–based models and other behaviors indicating antidepressant efficacy (e.g., learned helplessness, sucrose preference test) should also be explored. As our studies were designed to assess whether features of ketamine/ECS dosage paradigms could reveal new avenues for therapeutic interventions, we did not aim to determine the molecular mechanisms of ketamine’s metaplastic actions. Current evidence supports the necessity for NMDAR activity at the time of ketamine dosing such that sustained, antidepressant-relevant metaplasticity is detectable. This is supported by the SC-CA1 synapse not potentiating upon exposure to ketamine when slices were pretreated with an NMDAR antagonist (78). Furthermore, blocking the NMDAR at the time of ketamine injection prevented persistent antidepressant-relevant behavior and an increase in SC-CA1 LTP magnitude (20). Our results are consistent with these reports, and future studies are well positioned to elucidate the precise mechanisms of ketamine’s metaplasticity.

### Conclusions

Ketamine and ECT are among the most effective antidepressants but require repeated administrations for robust symptom relief. We discovered that ketamine administration evokes a metaplasticity that is harnessed by later doses to persistently promote the ability for synaptic potentiation. We used ketamine’s capacity to prime plasticity to reveal that subeffective doses of ketamine and ECS act synergistically to produce effective, persistent antidepressant-relevant behavioral and synaptic outcomes in the absence of cognitive impairment when delivered intermittently. Administration of an effective ketamine dose 24 hours before a single ECS also prolonged antidepressant-like actions. These results suggest that strategies harnessing metaplasticity can improve outcomes with available antidepressants and assist in identifying innovative interventions.

## Supplementary Material

1

2

3

4

Supplementary material cited in this article is available online at https://doi.org/10.1016/j.biopsych.2025.10.009.
