# Supplementary material for: Intermittent Ketamine and Neuromodulation Treatment Evoke Synergistic Antidepressant-Relevant Action
Source: Biol Psychiatry. Author manuscript; Available in PMC 2026 Jun 16. (PMC13270383; doi:10.1016/j.biopsych.2025.10.009)
Supplement: 1 [file NIHMS2184794-supplement-1.pdf]

SUPPLEMENTARY INFORMATION

Intermittent Ketamine and Neuromodulation Treatment Evoke Synergistic Antidepressant-Relevant Action

Brown and Gould

SUPPLEMENTAL FIGURES AND LEGENDS

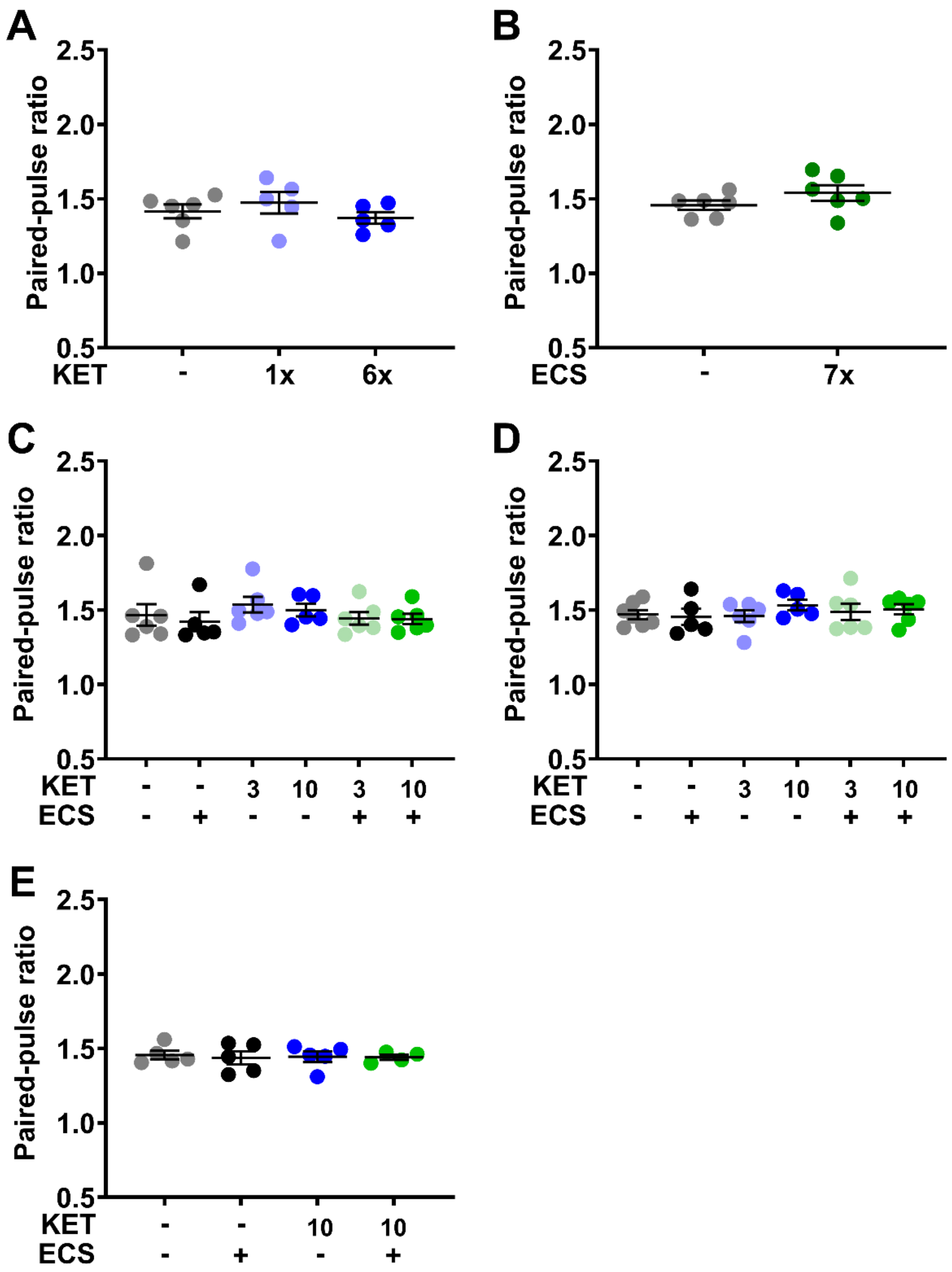

Supplemental Figure S1. Quantification of paired-pulse ratio (PPR) from treatment conditions. Average PPR from slices derived from mice treated in conditions described in Figure 1 (**A**), Figure 2 (**B**), Figure 3 (**C**), Figure 4 (**D**), and Figure 5 (**E**). See **Table S1** for complete details on the statistical analyses and precise group sizes. Data are the mean ± SEM. ECS, electroconvulsive stimulation; KET, (*R*,*S*)-ketamine.

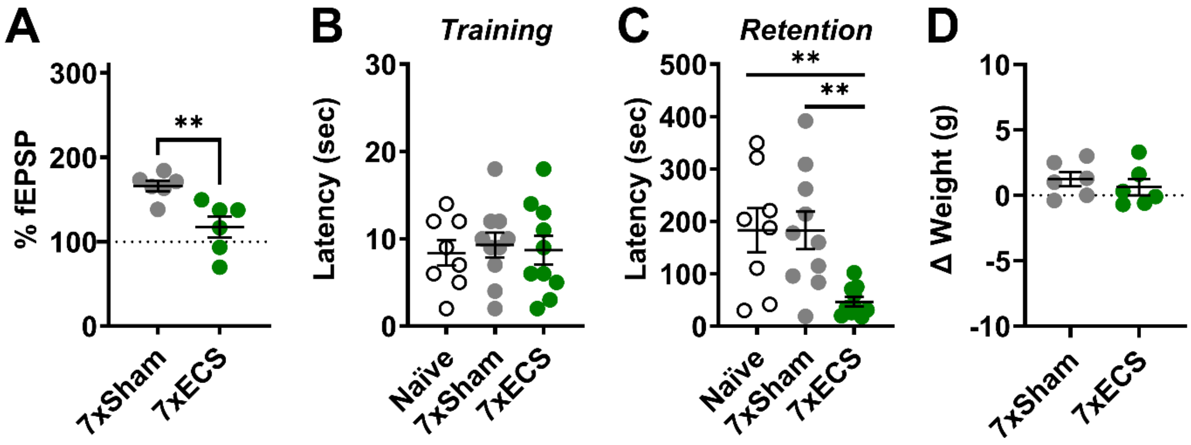

Supplemental Figure S2. Electrophysiology and behavioral data collected from naïve animals, animals treated with seven electroconvulsive stimulation (ECS) sessions, or animals treated with seven sham ECS sessions. (**A**) Summary plot of long-term potentiation magnitude measured from the hippocampal Schaffer collateral-CA1 synapse in slices derived from mice treated with seven sham ECS or seven ECS sessions. (**B-C**) Performance of naïve mice, mice treated seven times with sham ECS, and mice treated seven times with ECS in the training phase (**B**) and the retention phase (**C**) of the passive avoidance task. (**D**) The average change in weight of mice treated with seven sham ECS or seven ECS sessions. Data are the mean ± SEM. ** $p<0.01$ as indicated by unpaired Student's *t*-test (**A**) or Holm-Šídák *post-hoc* comparisons (**C**). See **Table S1** for complete details on the statistical analyses and precise group sizes. ECS, electroconvulsive stimulation.

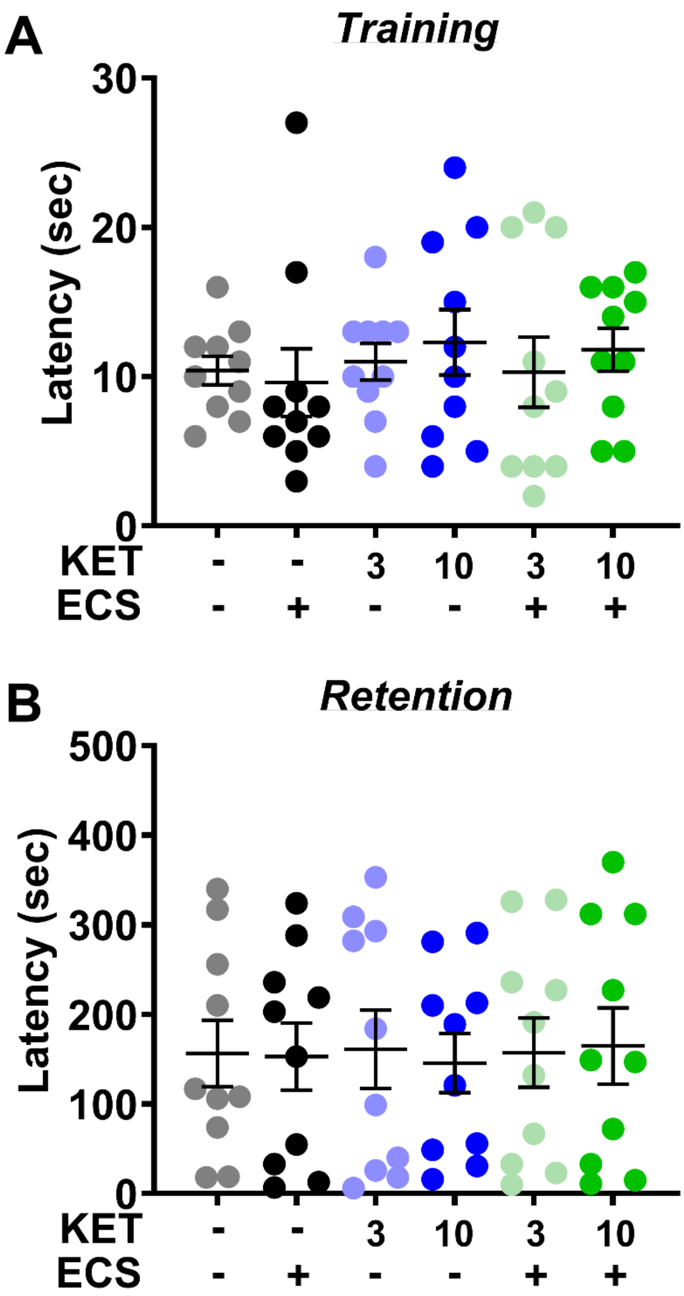

Supplemental Figure S3. Passive avoidance performance 24 hours after intermittent ketamine (KET) and electroconvulsive stimulation (ECS) treatment. The performance of mice in the training phase (**A**) and the retention phase (**B**) of the passive avoidance task following treatment as depicted in **Fig. 4A**. See **Table S1** for complete details on the statistical analyses and precise group sizes. Data are the mean ± SEM. ECS, electroconvulsive stimulation; KET, (*R*,*S*)-ketamine.
